# A Point Mutation in a Herpesvirus Polymerase Determines Neuropathogenicity

**DOI:** 10.1371/journal.ppat.0030160

**Published:** 2007-11-09

**Authors:** Laura B Goodman, Arianna Loregian, Gillian A Perkins, Josie Nugent, Elizabeth L Buckles, Beatrice Mercorelli, Julia H Kydd, Giorgio Palù, Ken C Smith, Nikolaus Osterrieder, Nicholas Davis-Poynter

**Affiliations:** 1 Department of Microbiology and Immunology, Cornell University, Ithaca, New York, United States of America; 2 Department of Histology, Microbiology and Medical Biotechnologies, University of Padua, Padua, Italy; 3 Department of Clinical Sciences, Cornell University, Ithaca, New York, United States of America; 4 Centre for Preventive Medicine, Animal Health Trust, Newmarket, United Kingdom; 5 Department of Biomedical Sciences, Cornell University, Ithaca, New York, United States of America; Oregon Health & Science University, United States of America

## Abstract

Infection with equid herpesvirus type 1 (EHV-1) leads to respiratory disease, abortion, and neurologic disorders in horses. Molecular epidemiology studies have demonstrated that a single nucleotide polymorphism resulting in an amino acid variation of the EHV-1 DNA polymerase (N752/D752) is significantly associated with the neuropathogenic potential of naturally occurring strains. To test the hypothesis that this single amino acid exchange by itself influences neuropathogenicity, we generated recombinant viruses with differing polymerase sequences. Here we show that the N752 mutant virus caused no neurologic signs in the natural host, while the D752 virus was able to cause inflammation of the central nervous system and ataxia. Neurologic disease induced by the D752 virus was concomitant with significantly increased levels of viremia (*p* = 0.01), but the magnitude of virus shedding from the nasal mucosa was similar between the N752 and D752 viruses. Both viruses replicated with similar kinetics in fibroblasts and epithelial cells, but exhibited differences in leukocyte tropism. Last, we observed a significant increase (*p* < 0.001) in sensitivity of the N752 mutant to aphidicolin, a drug targeting the viral polymerase. Our results demonstrate that a single amino acid variation in a herpesvirus enzyme can influence neuropathogenic potential without having a major effect on virus shedding from infected animals, which is important for horizontal spread in a population. This observation is very interesting from an evolutionary standpoint and is consistent with data indicating that the N752 DNA *pol* genotype is predominant in the EHV-1 population, suggesting that decreased viral pathogenicity in the natural host might not be at the expense of less efficient inter-individual transmission.

## Author Summary

Equid herpesvirus type 1 (EHV-1), a close relative of varicella-zoster virus and herpes simplex virus of humans, is spread by aerosol and is the causative agent of the most common neurologic disease of horses. Outbreaks of the neurologic form of EHV-1 can be devastating to individual animals and entire herds, and approximately one-third of the affected horses generally are at risk of death or suffer so extensively that euthanasia becomes necessary. Our report provides evidence for a direct causal link between the genotype of EHV-1 strains and their neurovirulence, and thereby gives a long-awaited explanation for the conundrum of the different clinical outcomes following EHV-1 infection. We proved that alteration of one amino acid in the key viral enzyme, DNA polymerase, which is conserved in all herpesviruses, renders the virus unable to cause neurologic disease. The improved clinical outcome is likely due to the reduction in virus levels in the bloodstream, ultimately resulting in less virus reaching the central nervous system. In summary, our study shows that herpesvirus virulence and tissue tropism in the natural host are linked with the function of a key virus-encoded enzyme involved in DNA replication.

## Introduction

Equid herpesvirus type 1 (EHV-1) is an aerosol-transmitted alphaherpesvirus, which causes rhinopneumonitis, abortion, and paralysis. Devastating outbreaks of the paralytic form of the disease occurred recently worldwide, resulting in its classification as a potentially emerging disease by the US Department of Agriculture [1]. A single nucleotide polymorphism in the catalytic subunit (Pol) of the viral DNA polymerase, causing a substitution of asparagine (N) by aspartic acid (D) at amino acid position 752, is significantly associated with outbreaks in which neurologic signs were recorded (*p* < 0.0001) [2].

Primary EHV-1 replication occurs in the respiratory tract, followed by spread to regional lymphatic tissues and dissemination via a cell-associated viremia [3,4]. In contrast to other neuropathogenic alphaherpesviruses, which cause encephalitis via neuronal infection, EHV-1-induced myeloencephalopathy is caused by infection of vascular endothelia of arteries supplying the central nervous system (CNS). The subsequent inflammatory response leads to thrombosis and ischemic damage [5,6]. A sustained and high-level presence of viral DNA in the blood stream, and, by implication, cell-associated viremia, is associated with the development of neurologic disease in EHV-1-infected horses [7,8]. The neurologic signs range from mild ataxia to complete paraplegia [9,10].

We hypothesized that mutation of the *pol* gene of a neuropathogenic strain to the N752 variant, which is rarely isolated from neurologic disease outbreaks, may cause a defect in cell-associated viremia, and, ultimately, less endothelial damage in the CNS vasculature. There are several possible mechanisms whereby Pol activity may influence the level of viremia, including (i) altered replication levels at the primary site of infection (respiratory epithelia), (ii) altered transmission to or levels of replication within circulating leukocytes, and (iii) altered efficiency of transmission from leukocytes to endothelial cells.

In the studies reported here, we confirmed the causal relationship between polymorphism in EHV-1 *pol* and neuropathogenicity in the primary host through targeted mutagenesis of a single nucleotide in the 150-kb DNA genome of the virus. The implication that this mutation directly affects the function of the Pol enzyme is further supported by our experiments with the Pol-targeting drug aphidicolin. Our data also indicate that the mutant and revertant viruses differ in their tropism for lymphocyte subsets, suggesting that the D→N752 mutation may affect replication in certain cell types.

## Results/Discussion

### BAC Cloning and Mutagenesis

**Figure 1:**
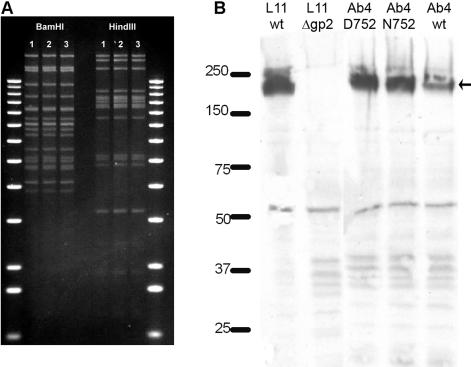
Viral Mutagenesis (A) Genomic restriction fragment patterns of the wild-type Ab4 strain (lane 1), the N752 mutant (lane 2), and the revertant D752 (lane 3) were analyzed to verify that no gross rearrangements had occurred during mutagenesis (marker, 1-kb ladder [Invitrogen]). (B) Expression of the glycoprotein restored in place of BAC sequences (gp2, 250 kDa, indicated by arrow) in all constructs was confirmed by Western blot analysis, with EHV-1 strain RacL11 (with and without gp2 [29]) and the parental Ab4 BAC as controls (marker, Precision Plus [Bio-Rad]). Mutant virus constructs were also sequenced in the region of interest at each passage level, including after reisolation from horses.

Bacterial artificial chromosome (BAC) cloning and mutagenesis facilitate manipulation of herpesvirus genomes [11] and is performed in Escherichia coli. Introduction of compensatory mutations is rare, because mutagenesis is independent of virus growth in eukaryotic cells [12]. Red recombination in E. coli allows introduction of point mutations without exogenous sequences being left behind [13]. We cloned neuropathogenic EHV-1 strain Ab4 at passage 8 as a BAC, via insertion of mini-F vector sequences into the nonessential gene 71 locus. During reconstitution of wild-type virus, as well as the mutant viruses used later in the study, gene 71 was repaired and vector sequences removed via homologous recombination in cells cotransfected with the EHV-1 BAC and a plasmid containing gene 71 sequences. We verified that the reconstituted, BAC-derived EHV-1 recombinants were identical to the parental isolate by restriction fragment length analyses and by Western blot analysis, which showed expression of gp2 encoded by gene 71 (Figure 1). Pilot studies showed that the BAC-derived and parental Ab4 viruses had similar in vitro growth properties and virulence in the BALB/c mouse model [14] as well as in ponies (unpublished data).

To test our hypothesis that the D752 Pol variant is responsible for enhanced, viremia-dependent inflammation of the CNS vasculature by neuropathogenic strains, we mutated the Ab4 BAC (D752) so that it would express the presumed non-neuropathogenic variant (N752) by two-step Red recombination [13]. Then, the N752 mutant was used to engineer a revertant virus (D752) by the same protocol. In all virus-positive lymphocyte cultures isolated from infected horses, we sequenced the region of interest in the mutant (N752) and revertant (D752) viral genomes at each stage of the experiment, to verify stability of the point mutation.

### Recombinant Viruses Have Similar Replication in Fibroblasts and in Mice

The D752 and N752 viruses had virtually identical single-step growth properties in equine NBL-6 dermal fibroblast cells (*p* = 0.6 and 0.8 for extra- and intracellular virus yields, respectively) at all tested time points post-infection (Figure S1). The ratios of intracellular genome copy numbers to plaque-forming units (PFU), averaged from samples collected at 8–36 h postinfection, were 1.1 × 10^4^ for the mutant, 4.1 × 10^3^ for the revertant, and 2.1 × 10^3^ for the parental BAC-derived virus (nonsignificant, *p* = 0.3). These data demonstrate no significant difference resulting from *pol* mutation in replication properties in vitro in equine fibroblasts.

The mutant N752 and revertant D752 viruses also caused similar daily body weight loss and viremia levels in groups of ten BALB/c mice each (Figure S2A and S2B). Six additional mice were infected per group, were humanely killed in the acute infection phase, and had similar viral loads in various tissues for both groups (Figure S2C). The mouse model of EHV-1 is a useful preliminary measure of attenuation. However, mouse brain microvascular endothelial cells are not susceptible to EHV-1, in contrast to those of horses [15]. Furthermore, the virus does not spread between mice, as it does in the natural host [16]. Our recovery of high numbers of viral genome copies from the brains and spinal cords of mice, in the absence of observable neuronal or endothelial damage in the CNS, highlights the deficiency of the mouse as a model for EHV-1 neuropathogenicity.

### The N752 Mutant Is Attenuated in the Natural Host

We conducted two infection trials in the natural host, first a pilot study in 2-y-old Welsh mountain ponies (four per group), and second a larger study in adult (3- to 16-y-old) mixed-breed horses (seven per group). Animals were randomly assigned to treatment groups and the identity of the treatments remained concealed from the investigators throughout the studies. In both experiments, virus was administered via the natural, intranasal route by aerosolization.

**Figure 2:**
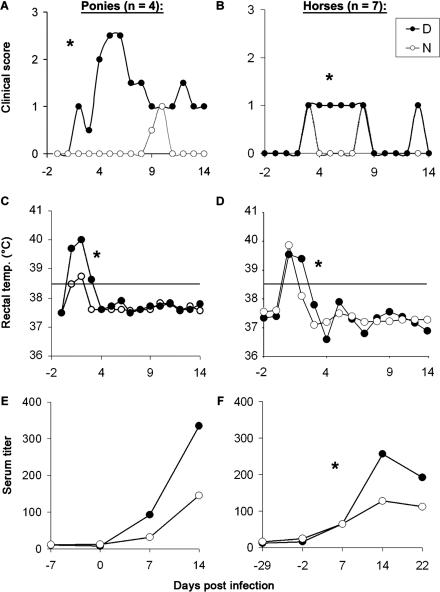
Clinical Data from the Equine Experimental Infection Studies Studies were conducted in a smaller pilot study (ponies) and subsequently a larger study (horses) with the revertant *pol* genotype virus (D752, •) and mutant virus (N752, ○). Data were collected before and after treatments. *p < 0.05. (A and B) Median cumulative clinical scores, which indicate overall symptom severity. (C and D) Median rectal temperatures (°C), with a line drawn at the cutoff temperature for fever (38.5 °C). (E and F) Median serum virus neutralizing antibody titers. Data from the first experiment on ponies (four animals/group) are shown in the left graphs (A, C, and E), those from the second experiment on horses (seven animals/group) in the right graphs (B, D, F).

**Figure 3:**
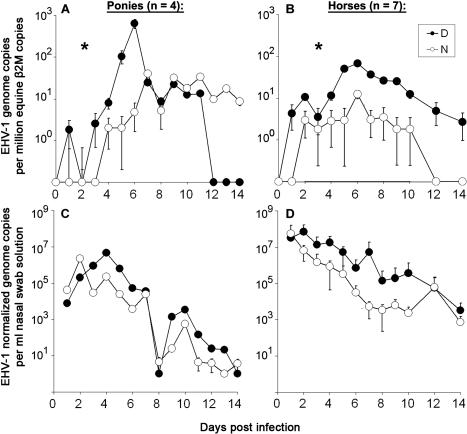
Real-Time Quantitative PCR Data from the Equine Experimental Infection Studies Results from studies conducted on ponies (A and C) and horses (B and D) with the revertant *pol* genotype virus (D752, •) and mutant virus (N752, ○). *p < 0.05. (A and B) Geometric means of normalized lymphocyte-associated viremia measured by qPCR, with EHV-1 DNA copies normalized per million cellular genomic DNA copies. (C and D) Virus titers in nasal excretions measured by qPCR. Shown are geometric mean normalized viral genome copies per milliliter of nasal swab solution. Standard deviations (error bars) are plotted, but are small compared to the y-axis scale and thus not visible for some data points. Data from the first experiment (ponies, four animals/group) are shown in the left graphs (A and C), those from the second experiment (horses, seven animals/group) in the right graphs (B and D).

**Figure 4:**
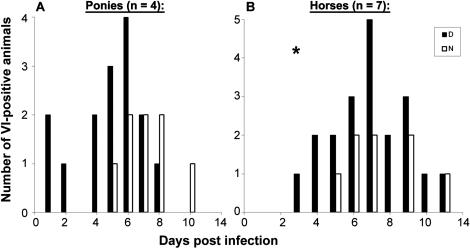
Viral Isolation from PBMC/RK13 Coculture Experiments Virus was isolated during the equine experimental infection studies with the revertant *pol* genotype virus (D752, •) and mutant virus (N752, ○). *p < 0.05. The number of culture-positive animals are shown, out of four total ponies per group (A) and seven total horses per group (B).

In the first study in ponies, clinical signs of upper respiratory tract disease, which comprised nasal discharge, coughing, and lymph node swelling, were more severe in the D752 (neuropathogenic genotype) virus group (Figure 2A). The cumulative clinical scores of the D752 group were significantly higher than those of the N752 group (*p* = 0.003). The D752 group also displayed higher median rectal temperatures during the first 3 d postinfection (Figure 2C), with a significantly longer duration of fever (median duration of temperatures >38.5 °C: 3 d for D752 and 1 d for N752; *p* = 0.01). Serum neutralizing antibody titers were similar between the groups (Figure 2E). Neurologic hind limb signs (mild ataxia) were observed in one pony from the D752 group and none from the N752 group, but detailed neurological assessments were not conducted in this study. Postmortem exams were performed on all ponies at 14 d postinfection. No specific signs of EHV-1 related pathology were detected in any of the ponies, but the spinal cords were not serially sectioned and hence focal pathology may not have been detected. Determination of viral load via quantitative PCR (qPCR) of peripheral blood mononuclear cells (PBMCs) demonstrated significantly higher titers (*p* = 0.03) in the blood for the D752 group, with maximum titers over 100-fold higher than in the N752 group (Figure 3A). Viremia was detectable, albeit at a low level, for a longer period for the N752 group, remaining at approximately ten copies per 10^6^ PBMC from days 12–14 postinfection, a time when titers were below the detectable threshold for the D752 group. Assessment of cell-associated viremia by virus isolation from heparinized blood (Figure 4A) detected viremia in all D752 ponies (median number of days positive: 4) compared with only two of the four N752 ponies (median number of days positive: 1.5). Peak levels and the kinetics of virus shedding determined by qPCR were similar for the two groups (Figure 3C), although on most days the D752 group had higher titers. The differences in virus nasal shedding, however, were not significant (*p* = 0.32).

Similar results were observed in the second study in older horses, which was conducted with larger animal numbers. The horses did not display the coughing or enlarged lymph nodes apparent in the pony infection study, but were positive for nasal discharge, with the D752 having a significantly higher nasal discharge severity score (*p* = 0.01) (Figure 2B). Rectal temperatures were also elevated for a significantly longer period for the D752 group (*p* = 0.009), with a median fever duration of 3 d for D752 and 1 d for N752 (Figure 2D). Serum neutralizing antibody titers were higher overall in the D752 group (Figure 2F). The peak viral load determined by qPCR in PBMC was approximately 10-fold higher for the D752 group (Figure 3B), with significantly higher mean titers recorded for the D752 group from days 1–14 postinfection (*p* = 0.01). Assessment of viremia by virus isolation also detected a significant difference in viremia (*p* = 0.04), with a median of 3 d positive in the D752 group and 0 d in the N752 group (Figure 4B). The peak levels and kinetics of viral DNA secretion were similar for the two groups (overall repeated measures *p* = 0.3, Figure 3D). However, analysis of the duration of nasal shedding of infectious virus using time to event analysis indicated that the D752 group had a longer duration (95% confidence interval for median duration: 3–5 d for N752 and 5–6 d for D752; *p* = 0.04).

The minimal infectious dose for EHV-1 is unknown, and we therefore do not know how much virus shedding is actually required for animal-to-animal transmission [17]. Although in both studies the titers for the D752 group were somewhat higher than those of the N752 group on most of the days sampled, the peak genome loads in nasal secretions of the two groups were similar, and there was no significant difference in overall virus shedding (Figure 3C and 3D). Although the duration of shedding can definitely not be discounted, and a longer duration of shedding of infectious virus was observed for the D752 group in the second study, we assume that the magnitude of shedding is a more important factor for transmission. By this measure, the two viruses were comparable in infectivity.

**Figure 5:**
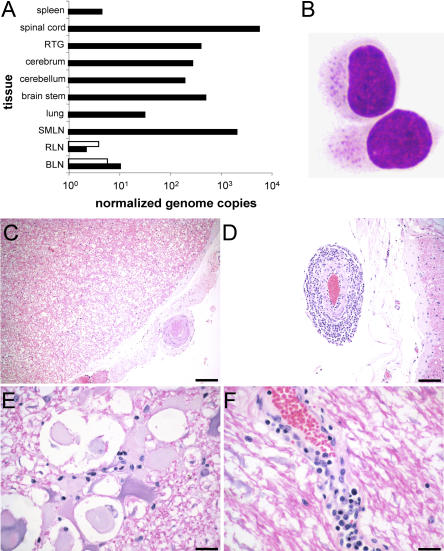
Postmortem Examination of Horses Infected with N752 or D752 Viruses (A) Normalized viral genome load in tissues collected from horses examined postmortem: N752–263 of the mutant group, with only two tissues positive for viral genome copies (□) and D752–4 of the revertant group (▪), with multiple positive tissues throughout the CNS and lymphatic system. RTG, right trigeminal ganglion; RLN, retropharyngeal lymph node; BLN, bronchial lymph node; SMLN submandibular lymph node. (B) Representative photomicrograph of lymphocytes present in the CSF of horse D752–4, with red cytoplasmic granules typical of cytotoxic T cells or natural killer cells. (C) Representative hematoxylin and eosin-stained section of caudal thoracic spinal cord tissue from horse N752–263 (bar 700 μm) showing no abnormalities. (D and F) Representative caudal thoracic cord sections from horse D752–4 (bars indicate, respectively, 1 mm, 40 μm, and 40 μm) showing lymphocytic cuffing in the meninges (D), dilated myelin sheaths with swollen axons, surrounded by reactive mononuclear cells (E), and typical lymphocytic perivascular cuffing (F).

We observed neurologic hind limb signs in two horses from the D752 group (Table 1). Because assessment of neurologic signs is a subjective parameter, neurologic exams performed during the larger trial were digitally recorded and reviewed by four independent equine specialists to provide diagnoses. Combining the results from the two studies, three of 11 animals showed neurological signs for the D752 group, compared with none of the 11 in the N752 group, although the difference in scores between the two groups was not significant (*p* = 0.06). We sampled and analyzed cerebrospinal fluid (CSF) to corroborate clinical observations. Figure 5B and Table 1 show data from CSF samples. Horse D752–4 was diagnosed with a neurologic grade of 2 (moderate hind limb ataxia; all four clinicians were in agreement), and horse N752–263 had no neurologic abnormalities. However, both horses had elevated nucleated cell counts and protein levels, indicative of inflammation (Table 1). Horse D752–4 had a CSF EHV-1 load two orders of magnitude higher than that of horse N752–263 (Table 1).

**Table 1:**
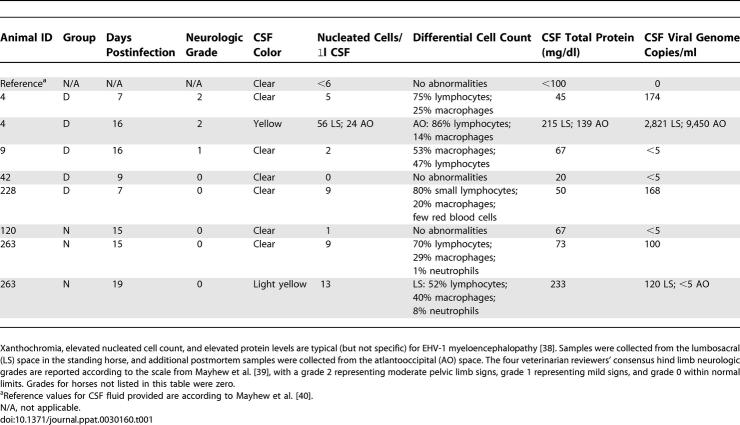
Neurologic Grades, CSF Cytology, and qPCR

Complete postmortem examinations on horses D752–4 (10-y-old female) and N752–263 (12-y-old castrated male) were conducted. Histological lesions in horse D752–4 were present throughout the meninges, but were most frequent and more severe in tissues overlying the caudal half of the thoracic cord as well as the cranial portion of the lumbar cord (Figure 5D–5F). Meninges showed moderate infiltration by lymphocytes, plasma cells, and scattered neutrophils, as well as substantial perivascular cuffing. Throughout the sections, veins and venules were dilated, congested, and lined by reactive endothelial cells. The spinal cord itself was largely unaffected, with the exception of two small perivascular lymphocyte cuffs and a focus of axonal swelling and dilated myelin sheaths in the white matter. In contrast, no CNS lesions were present in horse N752–263, which had the most severe illness among the horses in the mutant group (Figure 5C).

To confirm EHV-1 presence and to quantify viral load in tissues, we performed qPCR on a panel of lymphatic and CNS tissues collected postmortem (Figure 5A). Horse D752–4 had the highest viral load in the spinal cord at the L1 vertebral space. It also had EHV-1 genome copies in the brain, lung, and lymph nodes. In stark contrast, only tissue samples from two of the lymph nodes from horse N752–263 were qPCR-positive (Figure 5A).

### The D752 Neuropathogenic Virus Infects CD4^+^ Lymphocytes Preferentially

**Figure 6:**
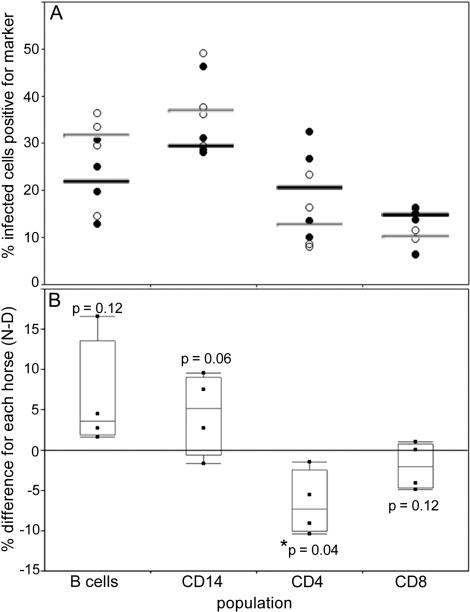
Equine PBMC Infection In Vitro (A) Each point represents the percentage of virus-infected cells that were positive for the respective equine cellular marker in an individual horse, as measured by FACS at 48 h postinfection. Cells were infected with either the neuropathogenic D752 revertant (•) or the non-neuropathogenic N752 mutant (○) virus; dark lines show median percentages for the revertant D752 group, and grey lines median percentages for the N752 mutant group. (B) Paired differences between the two viruses (percentage of infected cells expressing the respective cellular marker: N752 minus D752 for each individual horse) are plotted. The efficiency of PBMC infection ranged from 10% to 30%.

A reduction in cell-associated viremia may link viral replication and CNS inflammation [2]. We hypothesized that variation of the *pol* sequence may affect replication of EHV-1 in leukocytes. The horse experiment demonstrated this connection, particularly in the case of cells from CSF of horse D752–4, which typically exhibited cytoplasmic granules. This observation suggested the involvement of cells with a cytotoxic and/or natural killer phenotype in the inflammation within the spinal cord (Figure 5B). Therefore, we further hypothesized that the N752 mutant virus, in contrast to the D752 virus, may preferentially replicate or spread in certain cell types such as B cells or monocytes. We tested the hypothesis by infecting equine PBMCs in vitro with N752 or D752 viruses expressing enhanced green fluorescent protein (EGFP), and analyzing these cells by flow cytometry (Figure 6). When compared to the N752 mutant virus, D752 virus preferentially infected CD4^+^ cells (*p* = 0.04). There was a similar trend for increased D752 infection in CD8^+^ cells, but differences were nonsignificant (*p* = 0.12). Monocytes (CD14-positive) and B cells, however, showed the opposite trend (*p* = 0.06 for CD14 and 0.12 for B cells), as demonstrated by preferential infection of N752 in these subpopulations. These data suggest that the neuropathogenic variant has an enhanced tropism for the equine CD4^+^ T cell subpopulation in vitro. Further studies are needed to determine whether altered tropism for lymphocyte subsets is a major contributor to the more robust replication of D752 viruses observed in vivo, evidenced by higher levels of viremia (Figures 3A, 3B, and 4) and increased virus neutralizing antibody titers (Figure 2F). In particular, it will be important to compare the replication properties of the D752 and N752 recombinants in a variety of (primary) equine cell types.

### Increased Sensitivity to Aphidicolin of the N752 Virus and Polymerase Variant

**Figure 7:**
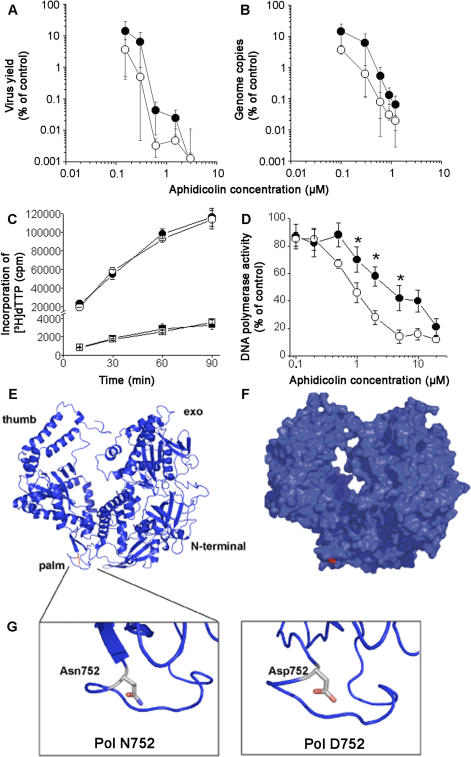
Differential Sensitivity of the Pol D/N752 Virus and Protein Variants to Aphidicolin and Structural Modeling of the Pol Region of Interest (A) Cells were infected with the Pol D752 revertant (•) or N752 mutant (○) virus, treated with aphidicolin, incubated 3 d, then lysed; final virus yield was titrated on new cells. (B) DNA was also extracted after the lysing step and qPCR performed to quantify normalized viral genome copies. (C) The DNA polymerase activity of Pol D752 and Pol N752 proteins, in the absence and in the presence of pORF18 (Pol accessory subunit), was analyzed by measuring the incorporation of [^3^H]dTTP into a poly(dA)-oligo(dT) template. (▪) Pol D752; (□) Pol N752; (•) Pol D752 + pORF18; (○) Pol N752 + pORF18. (D) The effect of aphidicolin on polymerase activity of Pol D752 (•) and of Pol N752 (○) was assayed by measuring the incorporation of [^3^H]dTTP into a poly(dA)-oligo(dT) template in the presence of pORF18. Graphs show the average of three experiments with standard deviations (error bars). Asterisk * indicates *p* < 0.05. (E) Ribbon diagram of EHV-1 Pol N752 is based on HSV-1 Pol crystal structure [23]. (F) Space-filling diagram highlights the region between HSV-1 Pol secondary structure elements P3 and PB on the outside surface of the palm domain. (G) Prediction of structural changes caused by the residue variation in Pol N752 as opposed to Pol D752.

The availability of polymerase-acting drugs provided the opportunity to investigate whether functional differences in vitro between the two EHV-1 Pol variants might exist and provide additional insight into the mechanism(s) underlying the different behavior of the viruses in vivo. Studies on human herpesvirus Pol mutants that exhibit altered sensitivity to drugs mimicking and/or competing with the natural substrates have helped to delineate enzyme regions and even single amino acid residues involved in catalysis and/or substrate binding [18]. The drug aphidicolin competitively inhibits the binding of certain deoxyribonucleoside triphosphates (dNTPs) to family B DNA polymerases, which include herpesvirus polymerases [19]. We performed virus yield titration and qPCR assays to evaluate the sensitivity in vitro of both the mutant N752 (non-neuropathogenic) and the revertant D752 (neuropathogenic) virus to aphidicolin. The N752 mutant virus was significantly more susceptible to the drug than the D752 revertant virus, with a reduction in 50% inhibitory concentration (IC_50_) of 33% (from 0.06 to 0.04 μM, *p* = 0.02) in virus yield titration assays (Figure 7A) and of 28% (from 0.07 to 0.05 μM, *p* < 0.0001) in qPCR assays (Figure 7B).

To investigate whether the observed differences in sensitivity to aphidicolin could be related to functional differences between the D752 and N752 Pol variants, we examined the DNA polymerase activity in vitro of both proteins in the absence or presence of the drug. As is the case in all herpesviruses studied to date, an accessory protein, EHV-1 pORF18, which stimulates nucleotide incorporation by Pol, forms the viral DNA polymerase holoenzyme together with the catalytic subunit [20]. Thus, we compared DNA polymerase activities of Pol D752 and Pol N752 in the absence or presence of pORF18 under different assay conditions (Figure 7C). No significant differences between the polymerase activities of Pol D752 and Pol N752, either in the absence (*p* = 0.85) or presence (*p* = 0.39) of pORF18, were observed. These results are consistent with the lack of differences in replication properties between the mutant N752 and the revertant D752 viruses seen in tissue culture experiments (Figure S1). We then investigated the in vitro effects of aphidicolin on polymerase activity of the two Pol variants. Pol N752 appeared more sensitive to aphidicolin than Pol D752 (Figure 7D), with an IC_50_ value of 0.9 versus 3.1 μM (*p* = 0.01). Again, these data agree with those obtained in cell culture (Figure 7A and 7B), although, in those experiments, differences in aphidicolin sensitivity were detected at lower drug concentrations. The differences in IC_50_ values between cell culture and DNA polymerase assays, which had also been observed in the case of a related herpesvirus Pol [21], may reflect the fact that multiple rounds of virus DNA replication in culture may help amplify aphidicolin's inhibitory effects.

To date, in contrast to a number of crystal structures of both prokaryotic and eukaryotic DNA polymerases (for a review see [22]), only one herpesvirus Pol structure has been reported, that of herpes simplex virus type 1 (HSV-1) [23]. The EHV-1 Pol variant amino acid (D/N752) is homologous to HSV-1 Pol residue D751. This position is located between highly conserved Pol regions II and VI [24], immediately following conserved motif A, which forms a portion of the dNTP binding cleft within the active site [23,25]. Structurally, this residue is on the outside of the palm domain (Figure 7E–7G), which houses part of the catalytic site of family B polymerases (e.g., eukaryotic Pol α and herpesvirus Pols) as well as that of family A polymerases (e.g., prokaryotic Pol I) [22]. Interestingly, conserved acidic (Asp and Glu) residues, found in close proximity to residue 752, have been identified in family B polymerases and are involved in metal-mediated interactions with the incoming dNTP [26]. We consider it likely that Pol residue 752 plays an important direct or indirect role in substrate binding and/or catalysis. This is because of (i) the high degree of conservation of an acidic residue (D or E) at the position equivalent to amino acid 752 of EHV-1 Pol in all herpesviruses [2], and (ii) the altered sensitivity to aphidicolin of the N752 variant when compared to D752 (Figure 7A–7D). Alternatively, the D to N change at position 752 might affect interaction(s) of residue 752 with amino acids involved in drug binding and catalysis by modifying Pol tertiary structure, and thereby result in altered drug sensitivity. The differences observed in structure predictions of the two Pol variant proteins (Figure 7G) lend support to this hypothesis. Subtle differences between the Pol enzyme variants in substrate binding and catalytic activity, not evident in rapidly dividing cell cultures in vitro, might become relevant in certain cell types in vivo, and thus influence the efficiency of lytic virus replication and/or virus reactivation from latency.

### Conclusions

The results of our experimental infection studies demonstrate that the N752 sequence variant of EHV-1 DNA Pol, when compared to the D752 variant, has reduced overall pathogenic potential and capacity to induce neurological signs. This reduced virulence is associated with lower levels of viremia, which is consistent with a previous study of EHV-1 field isolates suggesting that neuropathogenic strains exhibit higher levels of viremia [8]. Our study provides the evidence that DNA Pol sequence variation by itself has a significant effect on the overall level of viremia.

Leukocyte-associated EHV-1 viremia is believed to be an important aspect of the progression of infection to myeloencephalopathy, which is initiated by virus transfer from PBMCs to endothelia, relatively uninhibited by virus neutralizing antibodies. Following endothelial cell infection, inflammatory responses result in thromboischemic damage of neuronal tissue with neurological sequelae. We hypothesize that the D/N752 Pol sequence variation has a direct effect upon virus replication in certain cell types in vivo relevant to the development of cell-associated viremia. The demonstration of a subtle alteration of Pol activity, albeit apparent only as an altered sensitivity to the DNA Pol-targeting drug aphidicolin, is consistent with this hypothesis. Further studies are required to verify D/N752-related cell type-specific replication differences, but our studies demonstrating differences in relative efficiency of infection of different lymphocyte subsets in vitro, with D752 viruses having a preference for CD4^+^ T lymphocytes, may be relevant to the pathogenic potential of EHV-1, since these cells play key roles as virus carriers in the lytic and latent phase of the infection and in the inflammatory responses that result in EHV-1 disease.

Speculation as to the evolutionary origin of the D/N752 sequence variation for EHV-1 has been discussed previously [2]. In particular, the observation that all other herpesvirus polymerase sequences encode an acidic residue, generally aspartate, at the equivalent position to EHV-1 Pol 752, suggests strongly that the N752 variant arose from a D752 progenitor. Our estimate of the prevalence of the two Pol genotypes suggests that the majority (>75%) of EHV-1 strains encode N752 (unpublished data). Taken together, this information suggests that the N752 variant may have a selective advantage over D752, or at least no substantial disadvantage, for long-term maintenance within the equine population.

From our data it is reasonable to assume, therefore, that N752 viruses do not have a major defect in transmission. In the pony and even more so in the horse experiment, a more prolonged shedding of infectious virus for D752 than N752 was evident, which may potentially result in animals infected with D752 having a longer infectious period. It may be that the mucosal and systemic cellular immune response to N752 is more effective than that to D752, resulting in a shorter period of shedding. The observation of differences in the phenotype of leukocytes infected and the capacity of virus to replicate in these subpopulations suggest that further characterization of the cellular immune responses to these variants is worthwhile. However, the N752 virus was found to be shed in nasal secretions at levels similar to those of the D752 variant during the first 2 d postinfection, suggesting that initial rates of replication in the respiratory tract are similar. Since this period marks the peak titer of EHV-1 shedding, we postulate that the D/N752 variation has little effect, if any, on transmission when infected horses are likely to be at their most infectious. These results suggest that the N752 variant exhibits decreased disease severity and mortality without a substantial reduction of virus shedding and transmission, consistent with its higher prevalence. Other scenarios that might influence survival of either sequence variant in the population, such as potential effects on establishment of or reactivation from latency, are also conceivable, but remain to be tested.

In summary, we demonstrated that a naturally occurring variation in a single amino acid position of the viral DNA polymerase is responsible for differing pathogenic potential of a herpesvirus. In recent years, an increase in neuropathogenic outbreaks of EHV-1 has been reported, particularly in the US, although it is still controversial as to whether the increase is real or perceived due to heightened awareness. The confirmation that the EHV-1 Pol D/N752 sequence variation is directly associated with differences in pathogenic potential of individual virus strains provides a rationale for ongoing epidemiological studies, such as monitoring the current and past prevalence of the N752 and D752 genotypes. Such analyses will allow an adequate assessment of the risk of neurologic EHV-1 disease and help in designing efficient preventive and therapeutic measures for the most prevalent neurologic disease of horses.

## Materials and Methods

### Viral cloning and mutagenesis.

EHV-1 strain Ab4 (GB80_1_2 isolated from a quadriplegic mare [27]) was cloned as a BAC by cotransfection of a mini-F plasmid with wild-type genomic DNA in rabbit kidney RK13 cells, as described previously [28]. Briefly, the mini-F bacterial origin of replication sequence pHA2 containing a chloramphenicol resistance gene (*cat*) and the *egfp* gene were cloned into a transfer plasmid and introduced in lieu of nonessential gene 71 (encoding glycoprotein gp2 of EHV-1) by homologous recombination in RK13 cells [29]. The BAC was maintained in E. coli EL250 cells, which harbor the recombination system of phage λ under the control of a temperature-sensitive repressor [30]. The BAC was mutated to the opposite Pol genotype (N752) by converting nucleotide number 2254 of *pol* (*ORF30*) from a guanine to an adenine (see Table S1 for primers) using two-step Red-mediated en passant recombination [13]. Following confirmation of the respective genotypes by nucleotide sequencing (see Table S1 for primers), repaired virus was produced by cotransfection of a plasmid encoding EHV-1 Ab4 gene 71 with DNA of the respective mutant BAC. Resultant GFP-negative viruses, lacking the BAC cassette, were fully restored in gp2 expression, and exhibited restriction enzyme patterns identical to those of the parental Ab4 virus (Figure 1).

### Virus culture.

High-titer stocks of each virus were produced by passaging the transfection product once on equine NBL-6 cells in Eagle's minimal essential medium (EMEM) supplemented with 20% fetal bovine serum (FBS). Infected cells were frozen/thawed twice (−80 °C/37 °C), centrifuged for 5 min at 4,500*g*, supernatants were collected and stored at −80 °C. Titration of the virus suspension was performed on RK13 cells [29].

### DNA polymerase assays.

The pTM1-ORF30 plasmid, which expresses Pol (D752 variant) of EHV-1 Ab4 under a T7 promoter, was previously described [20]. The pTM1-ORF30 N752 plasmid harboring the *pol N752* mutant was created using the Quick-Change Mutagenesis kit (Stratagene, La Jolla, CA), amplifying the pTM1-ORF30 plasmid with primers listed in Table S1 pTM1-ORF30_D752N_F (forward) and pTM1-ORF30_D752N_R (reverse). The mutated *pol N752* gene was completely sequenced and shown to contain only wild-type sequences except for the engineered point mutation.

In vitro transcription-translation of the *pol D752* and *pol N752* genes was performed from plasmid pTM1-ORF30 or pTM1-ORF30 N752 using the TNT T7 coupled reticulocyte lysate system from Promega (Madison, WI) according to the manufacturer's guidelines. To examine protein expression levels, the translation products were labeled with [^35^S]methionine (Perkin Elmer, Waltham, MA) and analyzed by sodium dodecyl sulfate-7.5% polyacrylamide gel electrophoresis (SDS-PAGE) and autoradiography. Purified baculovirus-expressed pORF18, the accessory subunit of EHV-1 DNA polymerase, was prepared as described previously [20].

Basal DNA polymerase activity of Pol D752 and of Pol N752 and stimulation of their activity by pORF18 were assayed by measuring the incorporation of [^3^H]dTTP (Amersham Bioscience-GE Healthcare, Milan, Italy) into a poly(dA)-oligo(dT) template (Amersham Bioscience-GE Healthcare) as previously reported [20], using 12 μl of in vitro transcribed-translated Pol D752 or Pol N752 in the absence or in the presence of 600 fmol of purified baculovirus-expressed pORF18 in a 60-μl reaction volume.

The effect of aphidicolin on Pol activity was tested in similar assays, with 4 μl of in vitro-transcribed and -translated Pol D752 or Pol N752 plus 200 fmol of pORF18 in the presence or absence of various amounts of drug in a 20-μl reaction volume. Aphidicolin (Sigma-Aldrich, St. Louis, MO) was dissolved at a 300 μM concentration in 10% DMSO. In these assays, the final concentration of compound-derived DMSO was maintained at 0.5% (vol/vol) in all samples. In control samples with no drug added, a corresponding volume of pure DMSO was added to reach a final concentration of 0.5%.

### Viral drug sensitivity assays.

Aphidicolin (Calbiochem, San Diego, CA) was dissolved as a 1 mg/ml (2.95 mM) stock in DMSO, then serially diluted in EMEM supplemented with 0.5% FBS. For virus yield titration and qPCR assays, RK13 cells were plated at 2 × 10^5^ cells/ml in 24- and 96-well plates (BD Falcon, San Jose, CA), allowed to adhere overnight, and infected with N752 mutant or D752 revertant virus at a multiplicity of infection (MOI) of 0.01. After virus adsorption for 2 h at 37 °C, cells were washed twice with PBS and incubated with 1 ml (for virus yield titration assays) or 200 μl (for qPCR assays) of fresh media containing aphidicolin at various concentrations. Each final drug concentration was tested in three independent wells. The final concentration of the DMSO vehicle was less than 0.04 % (vol/vol). Plates were incubated for 2 d at 37 °C and then subjected to two freeze-thaw cycles (−80 °C and 37 °C). Virus yield titers were determined by transferring 100 μl aliquots from each of the wells to a fresh 24-well monolayer culture of RK13 cells followed by 1:10 serial dilution across the plate. Cultures were incubated for 3 d, then cells were stained, and the numbers of plaques were counted. Viral DNA genome copies were quantitated by qPCR as described below, using the cellular *18S rRNA* gene for normalization.

### In vitro PBMC infection assay.

PBMC were isolated by density gradient centrifugation over Histopaque 1077 (Sigma-Aldrich) from buffy coats of 30 ml of heparinized blood collected from healthy horses (approximate ages 10–29 y) with no history of ongoing or previous EHV-1 infection. Four experiments were conducted, each using blood from a different donor horse. Cells were infected immediately after isolation at an MOI of 1 using the GFP-expressing, reconstituted BAC clones of the Pol mutant (N752) and revertant (D752) viruses, and incubated for 48 h in conical polypropylene tubes (BD Falcon). Cell populations were characterized with a Becton Dickinson FACScalibur after immunofluorescent staining using mouse monoclonal antibodies recognizing either equine CD4 (clone CVS4, Abd-Serotec, Raleigh, NC), CD8 (clone 73/6.9.1, VMRD, Pullman, WA), or CD14 (clone 105, kindly provided by Dr. Bettina Wagner, Cornell University, Ithaca, NY), and a secondary Cy5-labeled anti-mouse antibody (Jackson ImmunoResearch, West Grove, PA), or a Cy5-conjugated goat-anti-horse B cell antiserum (kindly provided by Dr. Bettina Wagner).

### Animal experiments.

The mouse and horse experiments were performed with approval of the Cornell University Institutional Animal Care and Use Committee. The pony experiment performed in Newmarket (UK) was approved by the UK Home Office. All treatment administration, sample and data collection, clinical examinations, and statistical analyses for these studies were performed with the identity of the treatment concealed from the investigators.

BALB/c mice (*n* = 16 per group) were anesthetized with intraperitoneal xylazine/ketamine and inoculated intranasally with 1 × 10^5^ PFU of virus suspended in 25 μl of EMEM, or 25 μl of uninfected NBL-6 cell supernatant for the placebo group. Body weights were recorded daily and mean daily percentage body weight loss was compared between groups. Approximately 100 μl of blood was collected from ten mice per group at days 2, 6, and 9 postinfection by submandibular venipuncture, diluted to 200 μl with sterile 1× PBS containing 100 mM EDTA, and frozen at −80 °C. On days 3 and 5, three mice from each group were humanely killed by CO_2_ inhalation. Lungs, spleen, brain, and the upper 3–4 cervical vertebrae were collected using sterile procedures. Half of each organ was fixed in formalin for histopathology and immunohistochemistry; the other half was immediately frozen. DNA was purified in parallel with frozen blood samples using the DNeasy 96 Blood/Tissue kit (Qiagen, Valencia, CA).

Two independent equine infection experiments were performed. Both experiments tested the same BAC-derived mutant (N752) and revertant (D752) viruses. All animals enrolled had paired serum virus neutralizing antibody titers <1:32 (horses), or paired complement fixing antibody titers <1:10 (ponies) and were randomized to treatment groups. Starting 2 d prior to infection and for the duration of the experiment, animals were monitored daily for rectal temperature, nasal discharge, coughing, SMLN swelling, and neurologic signs. Cumulative clinical scores were calculated as follows: 1 point for serous nasal discharge, or 2 points for mucopurulent or heavy, discolored discharge; 1 point for infrequent coughing, or 2 points for frequent coughing; 1 point for SMLN swelling; 1 point for respiratory rate > 30, or 2 points for respiratory rate > 50.

Blood was collected daily in heparinized Vacutainers (Becton-Dickinson, San Jose, CA). Each buffy coat from two heparinized blood tubes was layered onto a gradient of Histopaque 1077 and 1119 for isolation of viable PBMCs as recommended by the supplier (Sigma procedure 1119). Serum from coagulated blood drawn at weekly intervals was assayed for viral neutralization by a veterinary diagnostic laboratory. Nasal swabs were also collected daily, by simultaneously inserting two 15-cm polyester-tipped swabs (Fisher Scientific, Pittsburgh, PA) into the ventral meatus of one nostril. The samples were immediately placed in 2 ml of viral transport media: 10% neonatal calf serum in phosphate-buffered saline, containing 3× antibiotic-antimycotic (Gemini BioProducts, Woodland, CA): 300 U/ml penicillin, 300 μg/ml streptomycin, 0.75 μg/ml fungizone; and 68 μg/ml enrofloxacin (Bayer Animal Health, Shawnee Mission, KS). Swabs were then incubated on ice for 2–4 h. For viral isolation, 200 μl of the nasal swab solution was diluted in EMEM (supplemented with 10% FCS and 3× antibiotic-antimycotic) and titrated on confluent monolayers of RK13 cells. After 2 h, the medium was replaced with 0.8% methylcellulose dissolved in the same growth medium. Plates were read 3 d postinoculation after acetone fixation and crystal violet staining. Results were recorded as titers of PFU per milliliter of inoculate. Another 200 μl of nasal swab solution was frozen at −80 °C and later thawed for DNA extraction as described below.

In the first equine experiment, two groups of four Welsh Mountain ponies, all 2-y-old females, were infected while housed in a BSL-3 facility in Newmarket, UK. Each group was in a separate room under negative air pressure with its own air supply. Each pony was given 1 × 10^7^ TCID_50_ (approximately 7 × 10^6^ PFU) of aerosolized virus, as described previously [31]. A second equine challenge experiment was performed at Cornell University, Ithaca, NY. Two groups of seven adult, mixed-breed horses, aged 3–16 y, were infected with 1 × 10^7^ PFU of aerosolized virus while housed in individual BSL-2 rooms as described previously [7]. Ages were normally distributed within each group, with a mean of 8 y in the D752 group and 10 y in the N752 group. Each group had five females and two castrated males. Horses were kept inside the rooms until virus could no longer be cultured from nasal swabs, then were led outside daily for neurologic exams. Under sedation with detomidine (Pfizer Animal Health, Exton, PA) at 0.01 mg/kg, CSF was collected aseptically as deemed necessary using a 5-in 18 gauge spinal needle in the lumbosacral space. CSF was also collected postmortem in the atlanto-occipital joint space from humanely killed horses. At necropsy, tissues from all major organs including brain and spinal cord were collected. Sterile tissue samples for qPCR were taken, immediately frozen at −80 °C, and processed as described below. CNS tissues were fixed in neutral buffered formalin as a whole and gross examination was completed after the tissues were fully permeated by the preservative. After gross examination was completed, representative sections of CNS tissue were processed for histopathology.

### Real-time quantitative PCR.

Aliquots of 5 × 10^6^ PBMC, 200 μl of nasal swab, and postmortem tissues were processed with the QIAamp96 DNA blood/tissue kit (Qiagen), with a final DNA elution volume of 200 μl. qPCR was performed using the 7500-FAST real-time PCR system (Applied Biosystems, Foster City, CA) with reaction mixtures containing TaqMan Fast Universal PCR Master Mix, 900 nM primers, 250 nM probe, and 5 μl of DNA sample, in a 20 μl total volume. The thermal cycling program was: 20 s at 95 °C, followed by 40 cycles of 95 °C for 3 s and 60 °C for 30 s. Each sample was run in triplicate. All normalized viral genome copy numbers for animal samples were calculated by the relative standard curve method, with the BAC clone of Ab4 used as a viral standard, a BAC clone of equine chromosome ECA1 for horse genome copy quantification (kindly provided by Drs. R. Tallmadge and D. Antczak [32]), and a partial clone of the murine *iNOS* (inducible nitric oxide synthase) gene for mouse genome quantification. A Taqman assay for the EHV-1 *IR6* gene was used to quantify viral genome copies [7]. Taqman genomic normalization assays were designed for the mouse *iNOS* gene or the horse β2-microglobulin gene, *β2-M* (Table S1). Normalization of viral DNA yield from nasal swabs was performed by addition of 5 × 10^3^ copies of Marek's disease virus (MDV) BAC clone pRB-1B [33] to the lysis buffer during DNA purification, and detecting copies of MDV gD [34]. Purification efficiency of nasal swabs was controlled by dividing each copy number result by the mean number of copies in all samples. Relative quantification of EHV-1 genome copies in the drug sensitivity experiment was performed using the ΔΔC_T_ method [35], with the *18S rRNA* gene as the endogenous control, and the untreated, infected well as the calibrator.

### Statistics.

Statistical analyses were performed using SAS v 9.1 (SAS Institute, Cary, NC). The significance level for all experiments was set at α = 0.05. Animal experiment data were fitted to linear models using PROC MIXED including appropriate two-way interactions, after verifying that residuals were normally distributed. Repeated ANOVA tests were performed for all experiments involving repeated sampling or repeated measurements. Significance of results was assessed at the pair-wise level using Bonferroni t-tests with α = 0.05. Data that did not pass a Shapiro-Wilks test for normal distribution of data are represented in figures in the form of medians or individual points.

Summary values were analyzed by Kruskal-Wallis testing. These values included: neurologic status (assessed as the highest score per horse), duration of fever (number of consecutive days of rectal temperature above 38.5 °C as measured each morning), and duration of infectious nasal shedding (number of days from the first to the last day of positive viral culture). Kaplan-Meier scores and p values for nasal shedding duration were calculated using PROC LIFETEST.

In vitro FACS raw data analysis was performed with FlowJo v. 7.2 (Tree Star, Ashland, OR), using the histogram function to measure the percent of infected cells expressing each cellular marker. Differences between virus treatments for PBMCs from different horses were tested for normality using the Shapiro-Wilks test, then compared to the null hypothesis of being equal to zero with a t-test. Differences between values in the in vitro aphidicolin sensitivity experiments were tested with a one-way ANOVA. IC_50_ values for the drug sensitivity assay were calculated based on linear regression model fit equations.

### Structure predictions.

Structure predictions were made based on the HSV-1 Pol crystal structure of Liu et al. as a template ([23], Protein Data Bank number 2GV9). The SwissProt accession numbers Q6S6P1 and P28858 were used for modeling Pol N752 and D752, respectively. The software program SWISS-MODEL (http://swissmodel.expasy.org/SWISS-MODEL.html) was used to make the diagrams [36]. In the surface rendering diagram, the solvent-accessible surface is blue and surface-exposed residues are red. All diagrams show the protein in the same orientation (annotated in Figure 7E).

## Supporting Information

Figure S1 Single-Step Growth Kinetics of D752 Revertant, N752 Mutant, and Parental D752 Ab4 Viruses in Equine Dermal Fibroblast NBL-6 Cells (5.0 MB PDF)

Figure S2 Comparison of Weight Loss, Viremia, and Viral Load in Tissues of BALB/c Mice Challenged Intranasally with 10^5^ PFU of N752 Mutant, D752 Revertant, Wild-Type Ab4 (wt) Virus, or Placebo (CTRL) (544 KB PDF)

Table S1 Oligonucleotides Used in This Study (12 KB PDF)

### Accession Numbers

The ORF30 (*pol*) sequence of the parental Ab4 virus is identical to that of GenBank (http://www.ncbi.nlm.nih.gov/) accession number AY665713. The structure predictions are based on SwissProt (http://www.expasy.org/sprot/) Q6S6P1 (N752) and P28858 (D752), using Protein Data Bank 2GV9 as a template.

## Abbreviations

BAC: bacterial artificial chromosome
CNS: central nervous system
CSF: cerebrospinal fluid
EHV-1: equid herpesvirus type 1
IC_50_: 50% inhibitory concentration
MOI: multiplicity of infection
ORF: open reading frame
PBMC: peripheral blood mononuclear cell
PFU: plaque-forming unit
Pol: DNA polymerase catalytic subunit
qPCR: quantitative real-time polymerase chain reaction
SMLN: submandibular lymph node
