# Supplementary material for: A Point Mutation in a Herpesvirus Polymerase Determines Neuropathogenicity
Source: PLoS Pathog. 2007 Nov 9;3(11):e160. doi: 10.1371/journal.ppat.0030160 (PMC2065875; doi:10.1371/journal.ppat.0030160)
Supplement: Supplementary file 1 [file ppat.0030160.sg001.pdf]

Figure S1

Single-Step Growth Kinetics of D752 Revertant, N752 Mutant, and Parental D752 Ab4 Viruses in Equine Dermal Fibroblast NBL-6 Cells

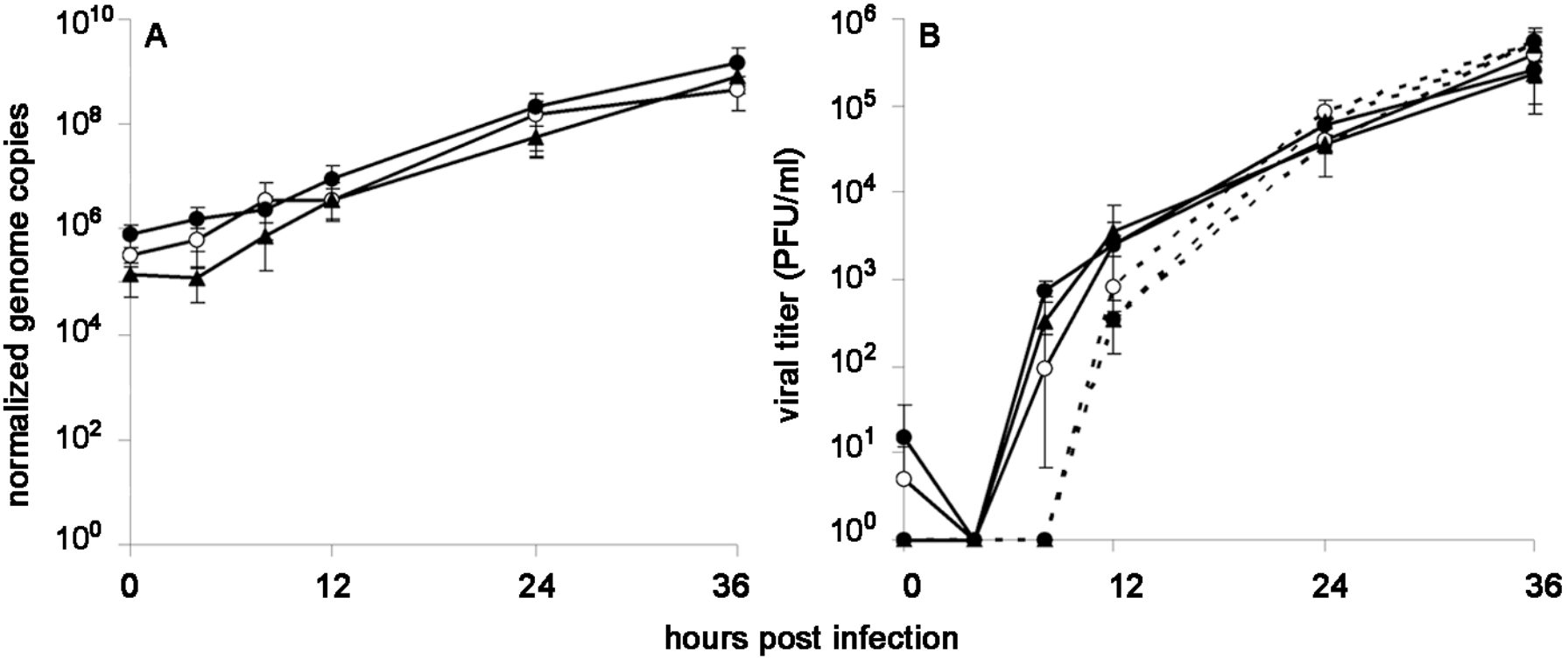

Key: D752 revertant (●), N752 mutant (○), and parental D752 Ab4 (▲). Cells were infected at an MOI of 5 and treated with a pH 3.0 buffer following viral penetration [37]. After washing, cells and supernatants were collected from separate wells every 4 h for 12 h, then every 12 h.

(A) Mean intracellular viral genome copies.

(B) Viral yield titers (solid lines are for intracellular titers, and dashed lines are for extracellular titers).
