# Supplementary material for: A Point Mutation in a Herpesvirus Polymerase Determines Neuropathogenicity
Source: PLoS Pathog. 2007 Nov 9;3(11):e160. doi: 10.1371/journal.ppat.0030160 (PMC2065875; doi:10.1371/journal.ppat.0030160)
Supplement: Supplementary file 2 [file ppat.0030160.sg002.pdf]

Figure S2

Comparison of Weight Loss, Viremia, and Viral Load in Tissues of BALB/c Mice Challenged Intranasally with 105 PFU of N752 Mutant, D752 Revertant, Wild-Type Ab4 (wt) Virus, or Placebo (CTRL)

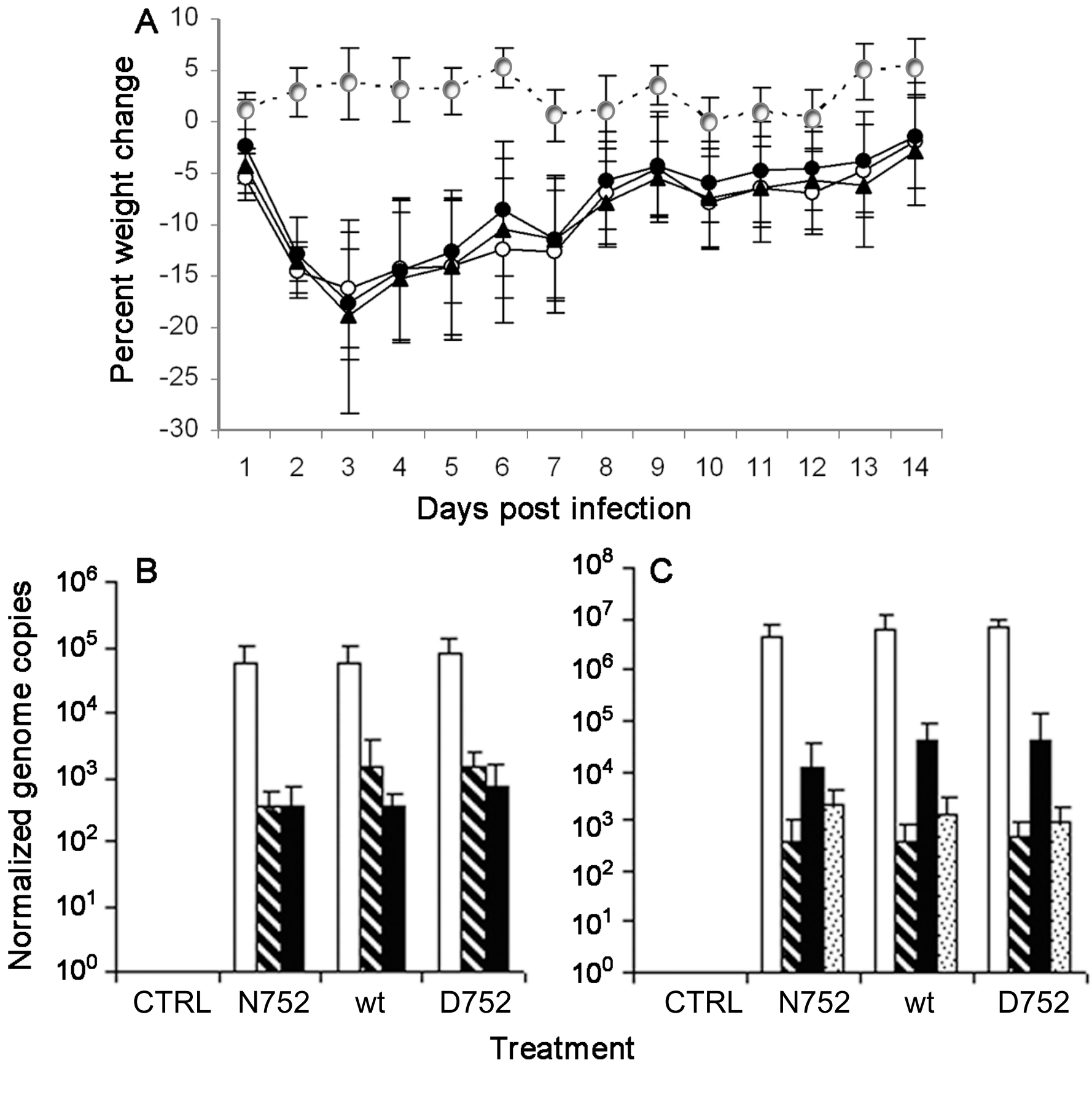

Weight loss (A), viremia (B), and viral load (C) in tissues are compared. Means and standard deviations (error bars) are shown in all panels. Weight loss is graphed as mean (n = 10) percentage change in body weight relative to baseline weight (— placebo, ● D752, ○ N752, ▲ wild-type). Viral load in blood and tissues was measured by qPCR and is plotted as EHV-1 genome (IR6 gene) copies per million mouse iNOS gene copies. Blood was collected on days 2 (open), 6 (hatched), and 9 (solid) postinfection by submandibular venipuncture in n = 10 mice per group. There was no difference in normalized copy numbers between nonplacebo treatment groups. Tissues were collected at days 3 and 5 postinfection from six mice in total per group (results were averaged over both days). Lungs (open), spleen (hatched), brain (solid), and the upper three or four vertebrae of the cervical spinal cord (dotted) were excised with sterile instruments. There was no difference between nonplacebo treatment groups for normalized EHV-1 genome copies in tissues.
