# Supplementary material for: A Point Mutation in a Herpesvirus Polymerase Determines Neuropathogenicity
Source: PLoS Pathog. 2007 Nov 9;3(11):e160. doi: 10.1371/journal.ppat.0030160 (PMC2065875; doi:10.1371/journal.ppat.0030160)
Supplement: Supplementary file 3 [file ppat.0030160.st001.pdf]

**Table S1. Oligonucleotides used in this study.**

For mutagenesis primers, the mutated nucleotide is in bold. The 3' ends of the primers (underlined) were designed to anneal to plasmid pEPkan-S, a plasmid used as a template for a PCR reaction that yields an amplification product containing an I-SceI site located upstream of the *aphAI* gene conferring kanamycin resistance (Tischer BK, von Einem J, Kaufer B, Osterrieder N (2006) Two-step red-mediated recombination for versatile high-efficiency markerless DNA manipulation in Escherichia coli. *BioTechniques* 40: 191-197). Sequencing primers for *pol* (*ORF30*) were first used to amplify a 466 bp fragment of *pol* from BAC DNA and also from virus co-cultured from equine PBMC and RK13 cells in the infection experiments. PCR products were purified using the Qiagen PCR cleanup kit prior to sequencing using the same primers. Oligonucleotides for measurement of murine *iNOS* and equine *β2-M* gene copies by qPCR were designed using Primer Express 2.0. The probes are fluorescently labeled on the 5' end with VIC, and have a non-fluorescent minor groove-binding (MGB) quencher.

| Purpose | Description | Sequence (5'-3') |
|---|---|---|
| mutagenesis | Ab4-*ORF30*_D752N_F | CGATGAAGTGGATCTGGCCGGGCTTCAACCATCCGTC**A**ACTACTCGACGTTCGAGTAGGGATAACAGGGTAATCGATTT |
| mutagenesis | Ab4-*ORF30*_D752N_R | AAATAACTTTTGGTCACCCACCTCGAACGTCGAGTAGT**T**GACGGATGGTTGAAGCCGCCAGTGTTACAACCAATTAACC |
| mutagenesis | Ab4-*ORF30*_N752D_F | CGATGAAGTGGATCTGGCCGGGCTTCAACCATCCGTC**G**ACTACTCGACGTTCGAGTAGGGATAACAGGGTAATCGATTT |
| mutagenesis | Ab4-*ORF30*_N752D_R | AAATAACTTTTGGTCACCCACCTCGAACGTCGAGTAGT**C**GACGGATGGTTGAAGCCGCCAGTGTTACAACCAATTAACC |
| mutagenesis | pTM1-*ORF30*_D752N_F | CAACCATCCGTCAACTACTCGACGTTCGAGG |
| mutagenesis | pTM1-*ORF30*_D752N_R | CCTCGAACGTCGAGTAGTTGACGGATGGTTG |
| sequencing | *ORF30*_F | GCTACTTCTGAAAACGGAGGC |
| sequencing | *ORF30*_R | CTATCCTCAGACACGGCAACA |
| qPCR | murine *iNOS*_F | CACTGTGTGCCAAGCCTAGGA |
| qPCR | murine *iNOS* probe | VIC-TCTGAGCGCCTCCAG-MGBNFQ |
| qPCR | murine *iNOS*_R | TCACAACGTGCCATCTGAAAG |
| qPCR | equine *β2-M* _F | TCTTTCAGCAAGGACTGGTCTTT |
| qPCR | equine *β2-M* probe | VIC-ATCTTCTGGTCCATACTGA-MGBNFQ |
| qPCR | equine *β2-M* _R | CATCCACACCATTGGGAG TAA A |
